# Reporting Characteristics of Cancer Pain: A Systematic Review and Quantitative Analysis of Research Publications in Palliative Care Journals

**DOI:** 10.4103/0973-1075.78451

**Published:** 2011

**Authors:** Senthil P Kumar

**Affiliations:** Department of Physiotherapy, Kasturba Medical College (Manipal University), Mangalore, India

**Keywords:** Cancer pain, Palliative care research, Reporting characteristics

## Abstract

**Objective:**

A common disorder requiring symptom palliation in palliative and end-of-life care is cancer. Cancer pain is recognized as a global health burden. This paper sought to systematically examine the extent to which there is an adequate scientific research base on cancer pain and its reporting characteristics in the palliative care journal literature.

**Materials and Methods:**

Search conducted in MEDLINE and CINAHL sought to locate all studies published in 19 palliative/ hospice/ supportive/ end-of-life care journals from 2009 to 2010. The journals included were: *American Journal of Hospice and Palliative Care, BMC Palliative Care, Current Opinion in Supportive and Palliative Care, End of Life Care Journal, European Journal of Palliative Care, Hospice Management Advisor, Indian Journal of Palliative Care, International Journal of Palliative Nursing, Internet Journal of Pain Symptom Control and Palliative Care, Journal of Pain and Palliative Care Pharmacotherapy, Journal of Palliative Care, Journal of Palliative Medicine, Journal of Social Work in End-of-life and Palliative Care, Journal of Supportive Oncology, Palliative Medicine, Palliative and Supportive Care, and Supportive Care in Cancer.* Journal contents were searched to identify studies that included cancer pain in abstract.

**Results:**

During the years 2009 and 2010, of the selected 1,569 articles published in the journals reviewed, only 5.86% (92 articles) were on cancer pain.

**Conclusion:**

While researchers in the field of palliative care have studied cancer pain, the total percentage for studies is still a low 5.86%. To move the field of palliative care forward so that appropriate guidelines for cancer pain management can be developed, it is critical that more research be reported upon which to base cancer pain therapy in an evidence-based palliative care model.

## INTRODUCTION

Reporting of scientific research in journals had been a topic of research for many years. Medical information was published initially in newspapers which later evolved into scientific journals.[1–3] Analysis of reporting characteristics provides the current status of research publications in journals. Reporting characteristics were reviewed previously and published in a variety of journals in the fields of general medicine,[4–23] dentistry,[24–26] and in secondary journals[27] that involved medical specialties such as anesthesiology,[28,29] dermatology,[30–34] emergency medicine,[35,36] endocrinology,[37] gastroenterology,[38] hepatology,[32] ophthalmology,[39,40] otorhinolaryngology,[41,42] physiology,[43] pediatrics,[44,45] pediatric dentistry,[46] pediatric psychology,[47] surgery,[48,49] veterinary medicine[50] and also in allied health,[51] nursing[52] and rehabilitation.[53]

Whilst some papers were on comparison between general and specialty journals on specific reporting characteristics,[54,55] few were on comparison between journals from a single database.[56] Most of the studies focused on reporting of ethical issues,[13–15,24,28,34,44,45,55] quality of reporting,[4–6,9–12,16–18,25,31,37–40,46,48,50,53] research methodology[7,19,20,32,33,35,36,41,45,47] and statistical issues,[8,21,22,26,29,43] very few studies were focused on reporting characteristics for clinical topics such as diagnosis,[30,38,40,52] and/or treatment[53,57] for diseases or disorders.[57–59]

Among the three papers that previously studied reporting characteristics specific to a patient population of cancer, one was on breast cancer,[57] one on Hodgkin’s lymphoma[58] while one was on a methodological aspect of reporting.[59] Vitry[57] identified the methodological shortcomings of medical intervention studies on patients with breast cancer and the author warned about exaggeration of therapeutic effects of many drugs due to inappropriate statistical methods of reporting in the reviewed clinical trials. Kober *et al*.,[58] in their paper studied the quality of reporting in clinical trials of patients with Hodgkin’s lymphoma where they compared the pre-CONSORT period with the post-CONSORT period and the authors found very few studies of high quality. Mathoulin-Pelissier *et al*.,[59] found inadequate reporting of survival end-points in randomized clinical trials of cancer in oncology journals.

Palliative care is a multidisciplinary profession and is being recognized as a separate field on its own. Evidence-based palliative care (EBPC) involved integrating effective research findings with clinical expertise and patient preferences towards better individualized provision of care for patients.[60] Evidence-based practice (EBP) in palliative care involves a step-by-step process of five distinct steps: formulation of research question, search for evidence,[61] critical appraisal of evidence,[62–64] implementation of evidence into practice, and outcome measurement. Often, the scientific rigor of systematic reviews had little or impact on a realistic clinical practice scenario to provide ‘high-level’ evidence.[65]

Clinical decision-making is a combination of art, statistics, experimentalism and EBP.[66] Thus evidence can be used to ‘inform’ current practice and it can further pave the way for development of suitable policy change.[67] Finding evidence for common clinical queries and presence of ‘conclusive’ evidence is always virtually impossible thus making application of evidence into practice a myth.[68] *Absence of evidence is often misunderstood as evidence of absence.* Evidence-informed practice (EIP) is an extended evolution of EBP in that it allows clinicians to apply their wealth of knowledge and experience and skills in the presence of ‘inconclusive or insufficient’ evidence.[69–73] However, it is also possible to integrate EBP and EIP in current practice,[74] and use of practice-based evidence established from qualitative studies also adds value in EIP.[75]

Cancer is a common clinical condition encountered in palliative care and pain is a common symptom addressed by healthcare professionals in a palliative care team. Cancer pain is no longer understood as a symptom, syndrome or a mechanism, it is a phenomenon.[76] The global burden of cancer pain and its impact on a patient’s quality of life is well understood. In palliative care, shared decision-making is often facilitated through presence of adequate evidence. Though a large amount of evidence existed for the prevalence of cancer pain,[77] and its under-treatment,[78] with equally large number of evidence-based practice recommendations and guidelines,[79–82] evidence for analysis of reporting characteristics on cancer pain was not found in the medical, oncological or palliative care literature.

Previously published studies on analysis of palliative care journals were on reporting of moral problems (ethical issues),[83] euthanasia,[84] chaplains and community-based clergy,[85,86] and religion and spirituality.[86–88] Thus there is a need to evaluate the reporting characteristics of cancer pain in palliative care journal literature in order to soundly implicate the establishment of evidence-based palliative care (EBPC).The objective of this paper was to perform a quantitative analysis of research articles on cancer pain published in palliative care journals in the years 2009 and 2010.

## MATERIALS AND METHODS

### Search strategy and criteria

Journals with names such as supportive, palliative, end-of-life, and hospice were included and searched from 2009 till 2010 for English abstracted papers in MEDLINE and CINAHL.

### Data synthesis

The total number of articles in all the selected journals was taken as N. The number of included articles (N_2_) based on search criteria were compared with number of articles that had ‘cancer and pain’ in abstract (N_1_) to obtain reporting rates (N_1_/N_2_%) for each journal. Such an estimate provided a gross reporting rate (GRR). Articles were later categorized as maximally related to cancer pain (studies where description was present as cancer pain; studies on cancer which included pain; studies on pain which also included cancer; and studies on cancer where pain was an outcome). The articles maximally related to cancer pain were termed as ‘mainly’ on cancer pain. The corrected reporting rates for individual journals were obtained by dividing this number of articles ‘mainly’ on cancer pain (N_3_) by total number of included articles (N_2_) from that journal. This estimate provided the corrected reporting rate (CRR=N_3_/N_2_%).

The journals were categorized broadly into MEDLINE-indexed and CINAHL-indexed. The reporting rates between MEDLINE-indexed and CINAHL-indexed journals were also compared for number of articles ‘mainly’ on cancer pain. Similarly, the reporting rates were compared for multidisciplinary, medical, nursing and other (social work) categories of palliative care journals. Comparison was also done for general versus cancer-specific palliative care journals.

**Figure 1:**
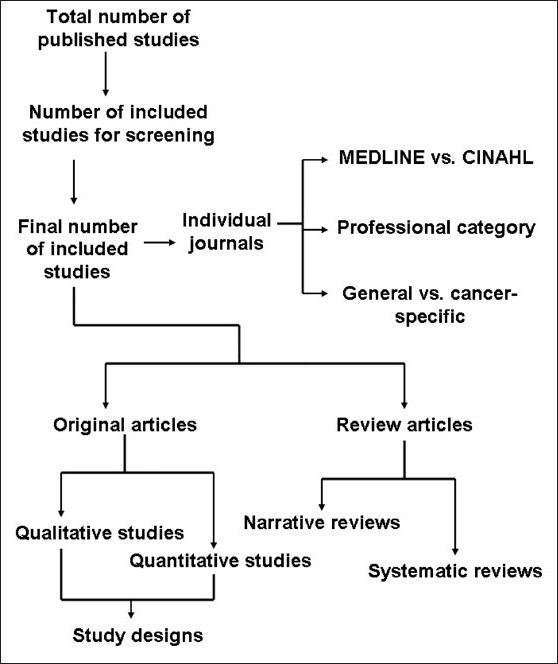
Schematic flowchart for data synthesis used in this study

The studies which were maximally related to cancer pain were then categorized into original articles and review articles. The original articles were then again grouped into qualitative and quantitative studies. Quantitative studies were then sub-grouped based upon study designs. The number of articles reported in each of the final subgroups was computed. The procedure of data synthesis is explained in the schematic flowchart [Figure 1].

### Data analysis

Descriptive analysis using frequencies for number of studies with respective percentiles was used for reporting characteristics and was done using 95% confidence interval by SPSS Version 11.5 (SPSS Inc, IL). Comparison between journals and article categories was done visually.

## RESULTS

### Overall journals’ characteristics

The study included 19 palliative care journals with a total number of 2600 articles. AJHPC- Am J Hosp Palliat Care; BMCPC- BMC Palliat Care; COSPC- Curr Opin Support Palliat Care; EOLCJ- End Life Care J; EJPC- Eur J Palliat Care; HMA- Hosp Manage Adv; IJPC- Indian J Palliat Care; IJPN- Int J Palliat Nurs; IJPSCPC- Internet J Pain Symptom Control Palliat Care; JPPCP- J Pain Palliat Care Pharmacother; JPC- J Palliat Care; JPM- J Palliat Med; JSWELPC- J Soc Work End Life Palliat Care; JSO- J Support Oncol; PCRT- Palliat Care Res Treat; PM- Palliat Med; PSC- Palliat Support Care; PPC- Progress Palliat Care; SCC- Support Care Cancer. Overall characteristics are outlined in Table 1.

**Table 1:** Overall journals’ characteristics

Total number of journals, N	19
MEDLINE-indexed/ CINAHL-indexed	12/7
Multidisciplinary/ Medical/ Nursing/ Other	15/2/1/1
General/ Cancer-specific	17/2
Supportive/ Palliative/Hospice/ End-of-Life	2/ 15/ 1/ 1

**Figure 2:**
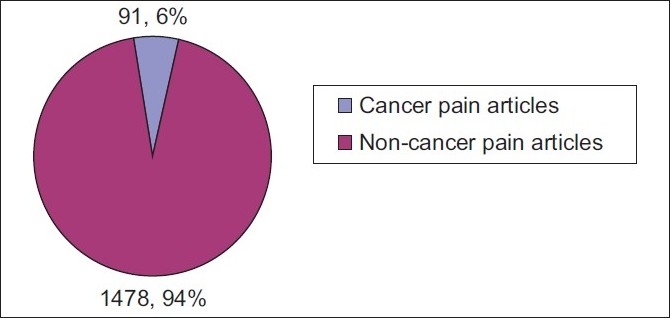
Overall prevalence of reporting cancer pain (corrected reporting rate) in all the palliative care journals

Out of a total of 1600 selected articles, 92 were on cancer pain with an overall reporting rate of 5.86% [Figure 2].

**Figure 3:**
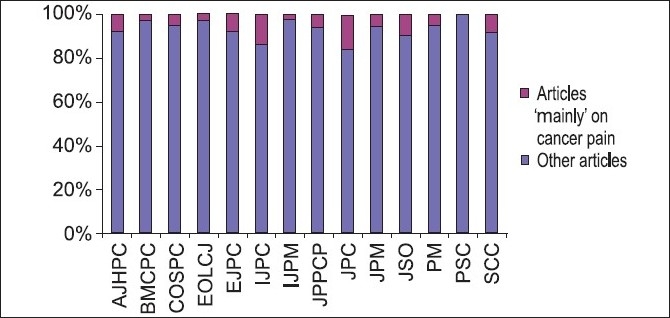
Comparison of reporting rates of articles ‘mainly’ on cancer pain-corrected reporting rate between palliative care journals

Individually, AJHPC had 10 articles,[89–98] BMCPC had one article,[99] COSPC had four articles,[100–103] EOLCJ had one,[104] EJPC had seven articles,[105–11] IJPC had five articles,[112–6] IJPN had two articles,[117,118] JPPCP had five articles,[119–23] JPC had six articles,[124–9] JPM had 13 articles,[130–42] JSO had four articles,[143–6] PM had eight articles[147–54] and SCC had 26 articles[155–180] ‘mainly’ on cancer pain. Also refer to Table 2 for respective reporting rates and to Figure 3 for comparison of number of ‘cancer pain’ articles and ‘non-cancer pain’ articles between the journals.

**Table 2:** Comparison of articles published in palliative care journals and their respective reporting rates of articles on cancer pain

	Total number of articles (2009-2010)	Articles in English with abstracts N_2_	Number of articles on cancer AND pain IN abstract N_1_	Gross percentage reporting N_1_/N_2_ % Gross reporting rate (GRR)	Number of articles ‘mainly’ on cancer pain N_3_	Corrected percentage of reporting N_3_/N_2_% Corrected reporting rate (CRR)
Am J Hosp Palliat Care^a^,^f^	180	137	15	10.94	10	7.29
BMC Palliat Care^a^,^f^	42	42	3	7.14	1	2.38
Curr Opin Support Palliat Care^a^,^f^	99	86	6	6.97	4	4.65
End Life Care J^b^,^f^	91	39	2	5.12	1	2.56
Eur J Palliat Care^b^,^f^	149	87	12	13.79	7	8.04
Hosp Manage Adv^b^,^f^	289	0	0	NA	NA	NA
Indian J Palliat Care^b^,^f^	70	38	8	21.05	5	13.15
Int J Palliat Nurs^a^,^d^	178	131	4	3.05	2	1.52
Internet J Pain Symptom Control Palliat Care^b^,^f^	16	13	0	0	NA	NA
J Pain Palliat Care Pharmacother^a^,^f^	113	84	7	8.33	5	5.95
J Palliat Care^a^,^f^	77	38	7	18.42	6	15.78
J Palliat Med^a^,^c^	470	246	22	8.94	13	5.28
J Soc Work End Life Palliat Care^a^,^e^	17	12	0	0	NA	NA
J Support Oncol^a^,^f^,^h^	87	42	6	14.28	4	9.52
Palliat Care Res Treat^b^,^f^	6	0	0	NA	NA	NA
Palliat Med^a^,^c^	199	161	16	9.93	8	4.96
Palliat Support Care^a^,^f^	118	101	3	2.97	0	0
Progress Palliat Care^a^,^f^	70	0	0	NA	NA	NA
Support Care Cancer^a^,^f^,^h^	329	312	40	12.82	26	8.33
Total number of articles, N or %	2600	1569	151	9.62	92	5.86

a - Medline-indexed journals;

b - Cinahl-indexed journals;

c - medical;

d - nursing;

e - other (social work);

f - multidisciplinary;

g - general;

h - cancer-specific;

NA - Not assessable since there were no available articles as per required criteria

**Figure 4:**
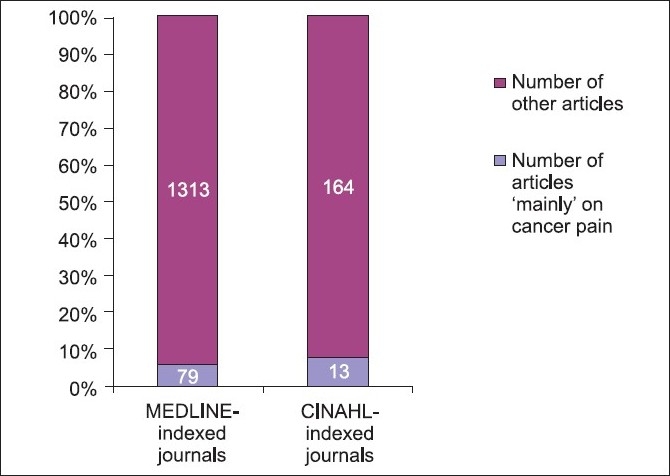
Comparison of corrected reporting rates between MEDLINE- and CINAHL-indexed journals

The first category included 12 journals indexed in MEDLINE: AJHPC, BMCPC, COSPC, IJPN, JPPCP, JPC, JPM, JSO, JSWELPC, PSC, PM and SCC. The second category included seven journals indexed in CINAHL: PCRT, EJPC, IJPC, IJPSCPC, PPC, ELCJ and HMA. The reporting rate for articles ‘mainly’ related to cancer pain in MEDLINE-indexed journals was 5.60% (78/1392) and in CINAHL-indexed journals was 7.34% (13/177) [Table 2 and Figure 4].

**Figure 5:**
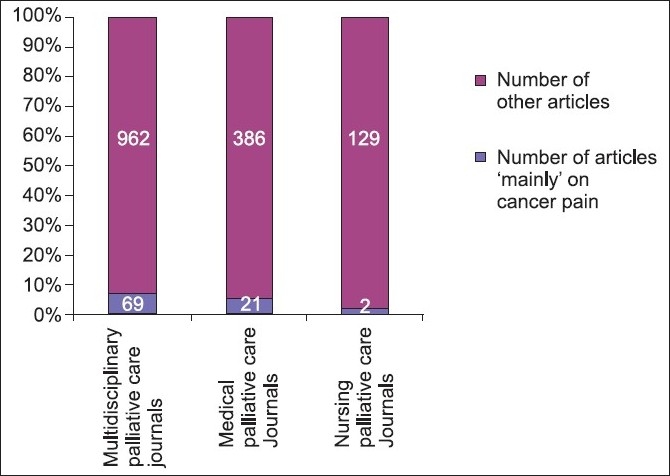
Comparison of corrected reporting rates between multidisciplinary, medical and nursing palliative care journals

The reporting rate for articles ‘mainly’ related to cancer pain in multidisciplinary journals was highest at 6.69% (69/1031) followed by medical journals at 5.15% (21/407) and one nursing journal at 1.52% (2/131). Also refer to Table 2 and Figure 5.

**Figure 6:**
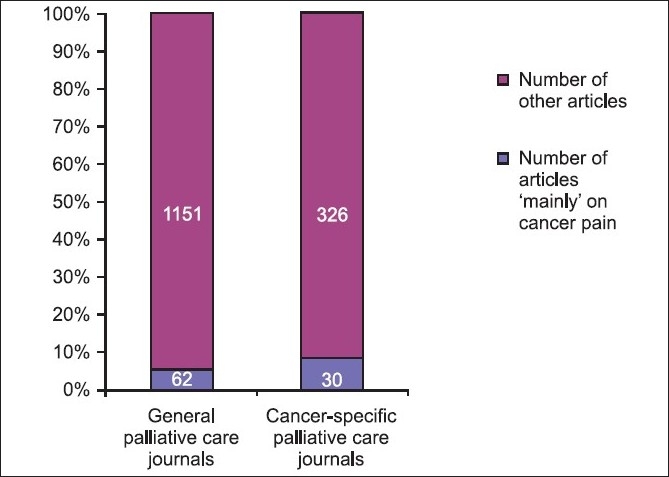
Comparison of corrected reporting rates between general and cancer-specific palliative care journals

All journals were focused on the general patient population except two-SCC and JSO which were cancer-specific and incidentally both were also under the ‘supportive’ name category. The two cancer-specific palliative care journals had a higher reporting rate of 8.42% (30/356) than general palliative care journals at 5.11% (62/1213). Also refer to Table 2 and Figure 6.

### Characteristics of ‘cancer pain’ articles

Of the 92 articles[89–180] on ‘cancer pain’, there were 68 original articles[89–99,104,105,112–7,121,123–8,130–8,140–3,146–52,154–7,159–63,165–75,177–9] and 24 review articles.[100–3,106–11,118–20,122,129,139,144,145,153,158,164,176,180] Among the original articles, there were 12 qualitative studies[115,116,118–20,122,124,126–8,155,169–71,173] and 56 quantitative studies.[89–99,104,105,112–4,117,121,123,125,130–8,140–3,146–52,154,156,157,159–63,165–8,172,174,175,177–9] There were eight randomized clinical trials,[94,143,150,157,161,162,167,177] 12 non-randomized clinical trials,[89,99,113,125,137,147,149,151,163,172,175,178] 11 cohort studies,[95,97,121,123,130,131,133,154,156,165,168] zero case-control studies, 10 cross-sectional studies[93,96,114,132,134,135,152,159,166,179] and 15 case reports[90–2,98,104,105,112,117,136,138,140–142,146,160] among the quantitative studies and there were two non-randomized clinical trials[115,147] one cohort study,[116] and nine cross-sectional studies[124,126–8,155,169–71,173] among the qualitative studies. There were six systematic reviews[129,153,158,164,176,180] and 18 narrative reviews[100–3,106–11,118–20,122,139,144,145] on cancer pain [Table 3].

**Table 3:** Characteristics of articles on cancer pain in terms of their type of article, method of research and study design

Total number of articles on cancer pain N_4_	Type of articles	Number of articles N_5_ (%= N_5_/N_4_)	Types of research methods	Number of articles N_6_ (%= N_6_/N_4_)	Study designs	Number of articles N_7_ (%= N_7_/N_4_)
92	Original articles	68 (73.91)	Qualitative studies	12 (13.04)	Randomized clinical trial	0
					Non-randomized clinical trial	2 (2.17)
					Cohort study	1 (1.08)
					Case control study	0
					Cross-sectional study	9 (9.78)
					Case report	0
			Quantitative studies	56 (60.86)	Randomized clinical trial	8 (8.69)
					Non-randomized clinical trial	12 (13.04)
					Cohort study	11 (11.95)
					Case control study	0
					Cross-sectional study	10 (10.86)
					Case report	15 (16.30)
	Review articles	24 (26.09)	Narrative reviews	18 (19.56)		
			Systematic reviews	6 (6.52)		

Figures in parenthesis are in percentage

### Evidence From Systematic Reviews And Randomized Clinical Trials Of Cancer Pain

The six systematic reviews[129,153,158,164,176,180] and eight randomized clinical trials[94,143,150,157,161,162,167,177] provided an evidence base as found from this review. The systematic reviews constituted 6.52% (6/92) and randomized clinical trials 8.69% (8/92) of the 92 cancer pain articles. But overall, they constituted meager reporting rates of 0.38% (6/1569) and 0.50% (8/1569) respectively for all journals combined for the years 2009 and 2010. The highest level of evidence (Level 1: Systematic reviews and randomized clinical trials) was thus reported in only 0.89% among all included articles and in 15.21% among those articles on cancer pain.

The two systematic reviews on assessments were on classification of cancer pain,[153] and orofacial pain due to cancer therapy;[158] and the four systematic reviews on treatments were on transdermal fentanyl,[129] intraspinal techniques,[164] massage therapy,[176] and cognitive effects of opioids.[180]

## DISCUSSION

This study is essentially the first of its kind to review palliative care journals utilizing a systematic approach to quantitatively identify reporting characteristics of articles on cancer pain. This study is the largest, and has included 19 palliative care journals. The previous authors, Hermsen and ten Have, reviewed 12 palliative care journals from 1984 to 1999,[83,84] found a reporting rate of 12% for ethical issues (458 articles) and the euthanasia rate was unreported (75 articles). Hermsen and ten Have[86] reviewed 12 journals from 1984-2002 and found a reporting rate of 2% for 80 articles on spirituality, pastoral care and religion. Flanelly *et al*.,[85] reviewed three palliative care journals from 1990-1999 and they found a reporting rate of 5.6% (47/838) for articles on the role of chaplains and clergy. The reason why this study found a smaller reporting rate could be due to the increased number of journals but this must have been counteracted by a shorter included duration of years of publication. This study included journals indexed in MEDLINE and CINAHL since they are the common databases for evidence search and this analysis of the last two years provided information on recent reporting rates.

The study found some interesting observations—some expected, some rather unexpected. The two expected observations include: Higher reporting rates among multidisciplinary palliative care journals since a ‘multidisciplinary’ focus for cancer pain had long been established in clinical palliative care practice, and, cancer-specific journals reported greater number of articles on cancer pain, more than other general palliative care journals. The unexpected observation was of higher reporting among CINAHL journals, but users should remember that though the *Indian Journal of Palliative Care* (IJPC) was included as a CINAHL journal as per the review date, the journal was indexed in MEDLINE, but not yet abstracted. Considering that IJPC was the second highest in reporting articles on cancer pain, this could change the review findings if performed at a later date.

The study was not aimed to perform a qualitative analysis or appraisal of the included articles (third step in EBP) since it was aimed more at finding the amount of existing evidence (second step in EBP). Lesser reporting rates may be attributed to already existing adequate research base for cancer pain, which again needs periodical updating for establishing the worthiness of the evidence since EBP emphasizes ‘current evidence’. Another area relatively less addressed is “refractory pain’ or “breakthrough pain”. Healthcare professionals need to be aware of the relatively lesser reporting of cancer pain in palliative care journal literature and should shoulder the responsibility to foster better number of reporting high-quality research on cancer pain. In future, such reviews could be performed with quality appraisal and identify the quality of reporting in cancer pain articles. Also, reviews on other related journals like oncology or cancer journals and anesthesia or pain journals may yield different results. This also opens a new area of debate on probable publication bias among certain journals which at present could not be studied or commented upon. Comparison of reporting characteristics between journals based on their specialty would direct clinicians to find research appropriate to answer their relevant clinical questions during EBPC.

## CONCLUSION

The overall prevalence in reporting of articles on cancer pain was low, only 5.86% among the 19 palliative care journals in this study reported ‘cancer pain’. Among the 19 palliative care journals, *J Palliat Care* ranked the highest to report articles which were mainly on cancer pain with a prevalence rate of 15.78%, followed by *Indian J Palliat Care* with 13.15% and J Support Oncol with 9.52%. The lowest reporting rate was found at 0% for Palliat Support Care. CINAHL-indexed, multidisciplinary and cancer-specific palliative care journals had a higher reporting rate than the MEDLINE-indexed ones, unidisciplinary, and general journals respectively. The systematic reviews constituted 6.52% (6/92) and randomized clinical trials 8.69% (8/92) of the 92 cancer pain articles. There is a need for better reporting of more research articles on cancer pain in palliative care journals.
